# Acetoacetate ameliorates skin fibrosis by modulating TGF-β1–Smad2/3 signaling pathway

**DOI:** 10.1016/j.jbc.2025.110867

**Published:** 2025-10-28

**Authors:** Ting Shang, Linxiao Li, Ke Fang, Xiaohui Miao, Jieshen Huang, Yu Jiang, Wuyan Lu, Zixin Cai, Yishu Lu, Lei Cui, Tinggang Chu, Hui Kang, Shuaijun Li, Jiefeng Huang

**Affiliations:** 1 Department of Stem Cells and Regenerative Medicine, Tongji University School of Medicine, Shanghai, China; 2 Department of Orthopedics, Shanghai Tenth People’s Hospital, Tongji University School of Medicine, Shanghai, China; 3 Mudanjiang Medical University, Mudanjiang, China; 4 Department of Plastic Surgery, Shanghai Tenth People's Hospital, Tongji University School of Medicine, Shanghai, China; 5 School & Hospital of Stomatology, Tongji University, Shanghai Engineering Research Center of Tooth Restoration and Regeneration, Shanghai, China; 6 Department of Plastic and Reconstructive Surgery, Affiliated Hospital of Nanjing University of Chinese Medicine, Nanjing, China

**Keywords:** skin fibrosis, ketone body, acetoacetate, Smad2/3 signaling pathway, OXCT1

## Abstract

Skin fibrosis is a progressive pathologic outcome of prolonged healing of cutaneous wounds, which has been well accepted as a metabolic disease in a recent study. However, the impact of ketone body metabolism on the development of cutaneous fibrosis remains largely unknown. Here, we found that ketone body metabolism was impaired in both human scars and bleomycin (BLM)-induced skin fibrogenesis of mice by bioinformatics analysis, which was further evidenced by downregulated expression of key modulators of ketone metabolism, including BDH1 (3-hydroxybutyrate dehydrogenase 1), OXCT1 (3-oxoacid CoA-transferase 1), and ACAT1 (acetyl-CoA acetyltransferase 1). With knockdown of OXCT1, a spontaneous onset of fibrosis in normal skin and exacerbation of BLM-induced skin fibrogenesis were observed. In dermal fibroblasts treated with transforming growth factor-beta 1, knockdown of OXCT1 improved their phenotype transition to myofibroblasts. Mechanistic studies indicated that phosphorylation of Smad2/3 signaling was markedly suppressed by acetoacetate supplementation. More importantly, we found that local administration remarkably alleviated fibrosis of BLM-treated skin in mice. Thus, our findings underscore the therapeutic potential of acetoacetate as an alternative intervention for skin fibrosis.

Skin fibrosis is a pathological result of a dysregulated progression in tissue repair and occurs in a variety of fibrotic disorders of the skin, such as scleroderma, hypertrophic scars (HSs), and keloids (1). Cutaneous injuries caused by burns, trauma, surgical incisions, and even insect bites can trigger skin fibrosis, which persists over a prolonged period, thus considerably affecting patients’ quality of life and mental status (2). However, current therapeutic strategies to attenuate skin fibrosis are limited and bring about a wide range of side effects, including skin atrophy, ulceration, and hyperpigmentation (3, 4). Therefore, there is an urgent need for exploring more effective therapies that can inhibit skin fibrosis.

In the fibrotic skin, dermal fibroblasts exhibit a significant enhancement in biosynthetic and proliferative activities, leading to an excessive deposition of extracellular matrix (ECM), including collagen and glycosaminoglycan (5, 6). Meanwhile, transforming growth factor-beta 1 (TGF-β1) plays a critical role in regulating ECM deposition by dermal fibroblasts through interaction with the classical Smad pathway (7, 8). In this context, the TGF-β1–Smad signaling pathway has been targeted to ameliorate tissue fibrosis (9).

Serving as a vital alternative metabolic fuel source, ketone bodies are transformed from acetyl-CoA produced from fatty acid β-oxidation in the liver (10). Acetoacetate (AcAc), β-hydroxybutyrate (β-OHB), and acetone are major types of ketone bodies, among which AcAc and β-OHB can convert to each other under the catalysis of β-hydroxybutyrate dehydrogenase (11). With the completion of their synthesis in the liver, ketone bodies are transported to extrahepatic tissues, such as the brain, muscle, and heart, for energy supply (12). In extrahepatic tissues, β-OHB is catalyzed into AcAc *via* BDH, which is subsequently converted to AcAc–CoA by the succinyl CoA-oxoacid transferase that is encoded by the nuclear Oxct1 gene. AcAc–CoA is then cleaved into two acetyl-CoA molecules by acetyl-CoA acetyltransferase 1 (ACAT1). The generated acetyl-CoA enters the tricarboxylic acid cycle and serves as fuel for ATP production.

Recently, the complicated relationship between metabolic processes, in particular the metabolism of ketone bodies and the development of fibrosis, has gained increasing interest. It was found that overexpression of 3-hydroxybutyrate dehydrogenase 1 (BDH1) in a failing heart showed a 1.7-fold increase in ketone body oxidation and significantly ameliorated heart fibrosis (13). An increased exogenous AcAc shuttle from hepatocytes to macrophages resulted in improvement in diet-induced hepatic fibrosis (14). However, the influence of ketone body metabolism on cutaneous fibrosis remains poorly described.

In this study, we examined the gene expression related to ketone body catabolism in fibrotic tissues from human keloids and bleomycin (BLM)-induced murine skin. Our findings indicated that the impairment in ketone body metabolism played a critical role in the pathogenesis of skin fibrogenesis. Furthermore, the supplementation of exogenous AcAc was shown to mitigate the progression of dermatofibrosis.

## Results

### Ketone metabolism remodeling is associated with skin fibrosis

**Figure 1:**
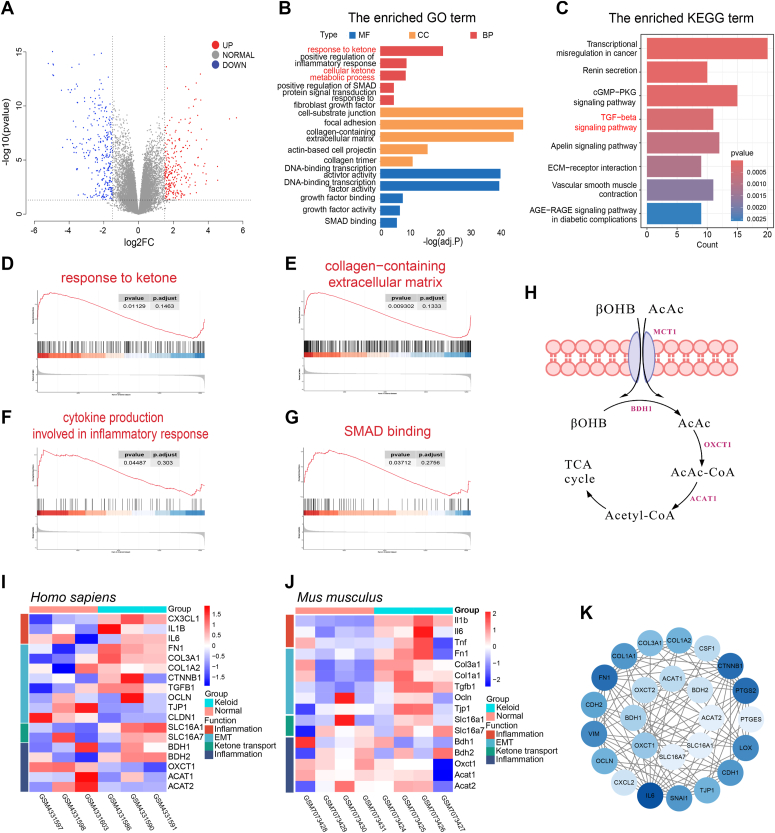
**Impaired ketone body metabolism is associated with skin fibrosis.***A*, Volcano plot showing differentially expressed genes (DEGs) in GSE145725. The upregulated genes (*red*) and the downregulated genes (*blue*) with *p* < 0.05 and logFC >1.5. *B*, Gene Ontology (GO) annotated statistics based on biological process (BP), cellular component (CC), and molecular function (MF) of DEGs between keloid and healthy skin. *C*, KEGG enrichment of relevant pathways. *D*–*G*, gene set enrichment analysis (GSEA) of response to ketones, collagen-containing extracellular matrix, cytokine production related to the inflammatory response, and SMAD binding from dataset GSE145725. *H*, schematic diagram of ketone body catabolism. *I*, heatmap showing DEGs between keloid and healthy skin. *J*, heatmap of DEGs between bleomycin-treated and healthy skin of mice. *K*, PPI network of DEGs related to ketone body metabolism and inflammation and fibrosis. FC, fold change; KEGG, Kyoto Encyclopedia of Genes and Genomes; PPI, protein–protein interaction.

To elucidate the connection between altered metabolic pathways and skin fibrosis, we conducted an analysis of *in vitro* cultured keloid-derived fibroblasts and normal dermal fibroblasts using the GSE145725 dataset. The result yields 568 differentially expressed genes (DEGs), comprising 261 upregulated and 307 downregulated genes, respectively. Variance analysis of those DEGs was presented in the volcano plot as shown in Figure 1*A*. By Gene Ontology enrichment analysis and Kyoto Encyclopedia of Genes and Genomes enrichment analysis, it was found that genes related to fibrogenesis, including cell–substrate junction, focal adhesion, collagen-containing ECM, positive regulation of Smad protein signal transduction, and the TGF-β signaling pathway, were upregulated in keloids (Fig. 1, *B* and *C*). More importantly, the analysis identified an increase in enrichment of ketone body metabolism, including response to ketones and cellular ketone metabolic processes in fibrotic keloid. The increase in response to ketones was further enriched by gene set enrichment analysis, along with significant enrichment in fibrogenesis, including collagen-containing ECM, cytokine production related to the inflammatory response, and SMAD binding in keloids (Fig. 1, *C*–*F*). This suggests a notable correlation between ketone metabolism and skin fibrosis. We thus analyzed RNA-Seq datasets of keloid-derived fibroblasts from keloid patients (GSE145725) and skin tissue from BLM-induced mice (GSE226331) (Fig. 1, *H*–*I*), respectively. RNA-Seq analysis unveiled decreased expression of genes involved in ketone catabolism, including BDH1, 3-oxoacid CoA transferase 1 (OXCT1), hydroxybutyrate dehydrogenase 2, ACAT1, and ACAT2, in the fibrotic skin of both humans and mice. Moreover, the association between ketone metabolism–related proteins, such as BDH1 and OXCT1, and fibrosis-related proteins was documented by the protein–protein interaction (PPI) network analysis (Fig. 1*J*). Collectively, these findings underscore a remodeling in ketone metabolism in pathological skin fibrogenesis.

### Ketone catabolism was impaired in fibrotic skin

**Figure 2:**
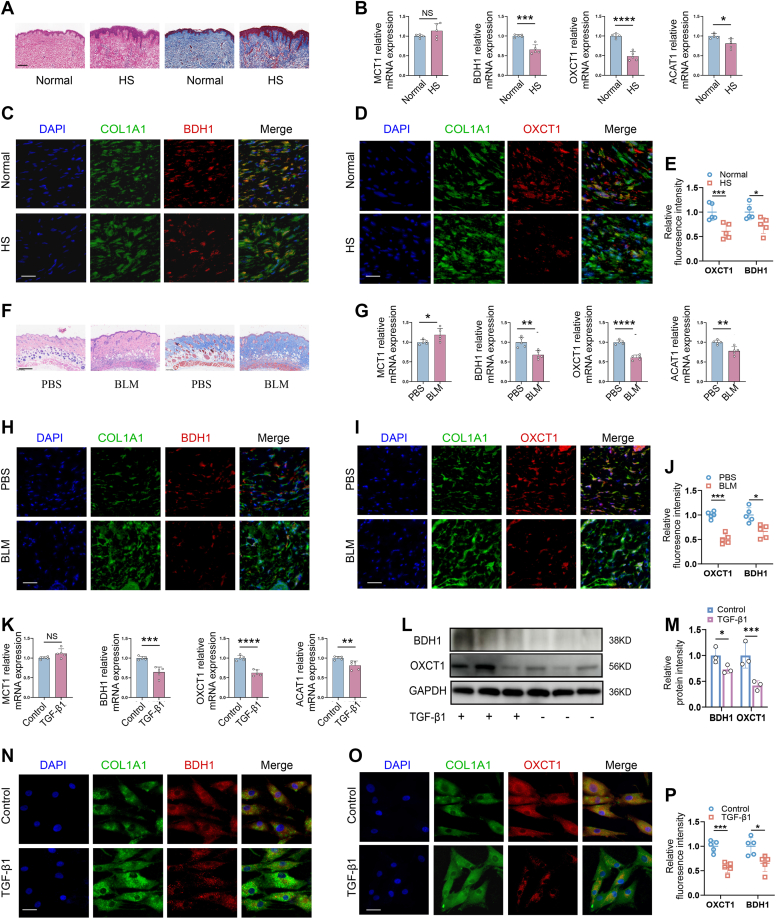
**Ketone body metabolism was impaired in fibrotic skin.***A*, H&E and Masson’s trichrome staining of hypertrophic scar (HS) and surrounding healthy skin samples. *B*, qRT–PCR of *Mct1*, *Bdh1*, *Oxct1*, and *Acat1* in HS and normal skin (n = 5 biological replicates per group). *C* and *D*, dual fluorescent staining images of BDH1 (3-hydroxybutyrate dehydrogenase 1) (*red*)/COL1A1 (*green*) and OXCT1 (3-oxoacid CoA-transferase 1) (*red*)/COL1A1 (*green*) in HS and normal. *E*, quantitative analysis of mean fluorescence intensity of BDH1 I and OXCT1 (*D*) (n = 5 biological replicates per group). *F*, H&E and Masson’s trichrome staining of bleomycin (BLM)-treated (5 mg/kg) and normal skin of mice. *G*, qRT–PCR of mRNA levels of *Mct1*, *Bdh1*, *Oxct1*, and *Acat1* in BLM-treated and normal skin (n = 5 biological replicates per group). *H* and *I*, representative images of dual staining of BDH1 (*red*)/COL1A1 (*green*) and OXCT1 (*red*)/COL1A1 (*green*) of BLM- and saline-treated skin at day 21 postadministration, respectively. J, quantitative analysis of mean fluorescence intensity of BDI (*C*) and OXCT1 (*D*). (n = 5 biological replicates per group). *K*, qRT–PCR of *Mct1*, *Bdh1*, *Oxct1*, and *Acat1* in human dermal fibroblasts treated with or without TGF-β1. *L*, Western blot of BDH1 and OXCT1 in human dermal fibroblasts stimulated with or without TGF-β1. *M*, semiquantitative analysis of Western blot for BDH1 and OXCT1. *N*–*O*, representative images of dual staining of BDH1 (*red*)/COL1A1 (*green*) and OXCT1 (*red*)/COL1A1 (*green*) in human dermal fibroblasts treated with or without TGF-β1. *P*, quantitative analysis of mean fluorescence intensity of BDH1 (*N*) and OXCT1 (*O*). (n = 5 biological replicates per group). All scale bars represent 50 μm. Data expressed as the mean ± SEM. Significant differences were determined by Student’s *t* test. ∗*p* < 0.05, ∗∗*p* < 0.01, ∗∗∗*p* < 0.001, and ∗∗∗∗*p* < 0.0001. qRT–PCR, quantitative RT–PCR; TGF-β1, transforming growth factor-beta 1.

Based on the analysis of the skin fibrosis database, we next investigated the expression of key regulators involved in ketone catabolism in the pathogenesis of skin fibrosis. In human HSs, which are characterized by increased fibrotic matrix deposition and epidermis thickness (Fig. 2*A*), expression of BDH1, OXCT1, and ACAT1 was significantly decreased compared with that in normal skin by quantitative RT–PCR (qRT–PCR). While expression of MCT1 showed no significance (Fig. 2*B*). Accordingly, the numbers of BDH1 (*p* < 0.05) and OXCT1 (*p* < 0.01)-positive cells were markedly reduced in human hyperplastic scar as shown by immunofluorescent staining (Fig. 2, *C*–*E*). In BLM-induced fibrotic mouse skin, downregulation of genes related to ketone body catabolism was also indicated by qRT–PCR assessment (Fig. 2, *F*–*J*). In addition, a significant decrease in the number of OXCT1- (Fig. 2*H*, *p* < 0.01) and BDH1-positive (Fig. 2*I*, *p* < 0.01) cells as well as in expression of OXCT1 and BDH1 was detected in the BLM-treated skin.

We next treated human dermal fibroblasts with TGF-β1 to examine changes in expression of key catalysts regulating ketone metabolism. We found that the levels of expression of OXCT1 and BDH1 were markedly decreased in TGF-β1-treated fibroblasts, as determined by Western blot and qRT–PCR, respectively (Fig. 2, *K*–*M*). Meanwhile, immunofluorescent staining showed that treatment with TGF-β1 resulted in a significant reduction in OXCT1- (*p* < 0.01) and BDH1- (*p* < 0.01) positive cells (Fig. 2, *N*–*P*). Together, these findings suggested that ketone catabolism was impaired in the pathogenesis of skin fibrosis.

### Knockdown of OXCT1 induced the onset and exacerbation of skin fibrosis

**Figure 3:**
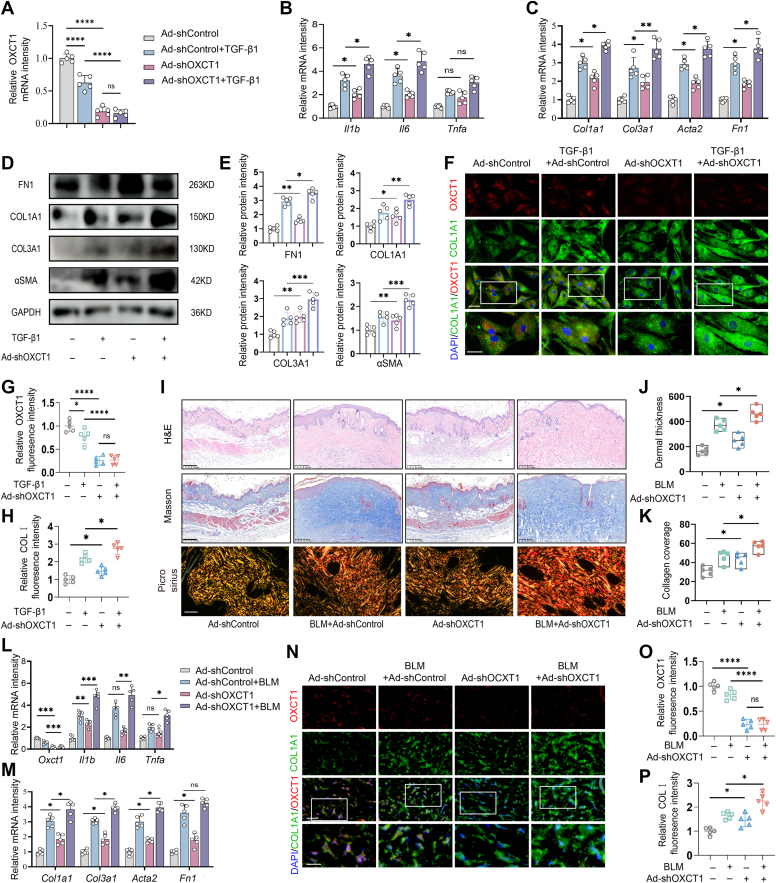
**Knockdown of 3-oxoacid CoA-transferase 1 (OXCT1) induced and exacerbated skin fibrosis.***A*, real-time RT–PCR of OXCT1 in human dermal fibroblasts transfected with Ad-shOXCT1 for 48 h, in the presence or absence of TGF-β1 (5 ng/ml). *B* and *C*, expression of fibrosis-associated (*Col1a1*, *Col3a1*, *Acta1*, and *Fn1*) (*B*) and inflammation-related genes (*Il6*, *Il1b*, and *Tnfa*) (*C*) in fibroblasts transfected with Ad-shOXCT1. (n = 5 biological replicates per group). *D* and *E*, Western blot of α-SMA, COL1A1, COL3A1, and FN1 in cells with OXCT1 knockdown. GAPDH served as a loading control. Semiquantitative analysis of Western blot for FN1, COL1A1, COL3A1, and αSMA. *F*, representing images of dual fluorescent staining of OXCT1 (*red*) and COL1A1 (*green*). The scale bar represents 50 μm, and the lowest images are enlarged insets of the *upper panel* (the scale bar represents 200 μm). *G* and *H*, quantitative analysis of mean fluorescence intensity of OXCT1 (*G*) and COL1A1 (*H*). (n = 5 biological replicates per group). *I*, representative images of H&E, Masson’s trichrome, and Sirius red staining of bleomycin (BLM)-treated skin with or without knockdown of OXCT1. The scale bar represents 200 μm. *J* and *K*, semiquantitative analysis of dermal thicknesses and collagen coverage in skin using ImageJ. (n = 5 biological replicates per group). *L* and *M*, real-time RT–PCR of OXCT1, inflammation-associated genes (*Il6*, *Il1b*, and *Tnfa*), and fibrosis-associated genes (*Col1a1*, *Col3a1*, *Acta1*, and *Fn1*), with β-actin as an internal control. (n = 5 biological replicates per group). *N*, immunofluorescence images of OXCT1 (*red*) and COL1A1 (*green*) in BLM-challenged skin. The scale bar represents 50 μm, and the lowest images are enlarged insets of the *upper panel* (the scale bar represents 200 μm). *O* and *P*, quantitative analysis of mean fluorescence intensity of OXCT1 and COL1A1. (n = 5 biological replicates per group). Data expressed as the mean ± SEM. Statistical significance was analyzed using two-way analysis of variance with multiple comparisons. ∗*p* < 0.05, ∗∗*p* < 0.01, ∗∗∗*p* < 0.001, and ∗∗∗∗*p* < 0.0001. α-SMA, α-smooth muscle actin; FN1, fibronectin 1; TGF-β1, transforming growth factor-beta 1.

Given that ketone catabolism was impaired in the pathogenesis of skin fibrosis, we addressed whether dysfunction of ketone body metabolism induced by knockdown of OXCT1 would exacerbate skin fibrosis. Expression of OXCT1 was significantly decreased by transfection of adenovirus-mediated siRNA against OXCT1 in human dermal fibroblasts (*p* < 0.01). Treatment with TGF-β1 induced no more enhancement in OXCT1 expression in Ad-shOXCT-transfected cells (Fig. 3*A*). In TGF-β1-treated fibroblasts, Ad-shOXCT1 transfection induced a significant increase in expressions of COL1A1, COL3A1, fibronectin, and α-smooth muscle actin (α-SMA), at both mRNA and protein levels, in comparison to that treated with TGF-β1 alone, as revealed by qRT–PCR (Fig. 3*C*) and Western blot analyses, respectively (Fig. 3, *D* and *E*). The enhancement of COL1A1 expression resulting from Ad-shOXCT1 transfection was further manifested by immunofluorescence analysis (Fig. 3, *F*–*H*). It is noteworthy that transfection of Ad-shOXCT1 alone induced a remarkable increase in fibrogenesis of dermal fibroblasts. Meanwhile, we found that expression of proinflammatory cytokines, interleukin (IL)-6 and interleukin-1, was significantly elevated in Ad-shOXCT1-treated fibroblasts, in the presence or absence of TGF-β1, compared with those in Ad-shControl-treated control fibroblasts (Fig. 3*B*).

We next investigated whether OXCT1 knockdown would enhance skin fibrogenesis *in vivo* by subcutaneously injecting Ad-shOXCT1 (1 × 108 pfu) in mice that are treated with BLM (5 mg/kg) once a week for 8 weeks. Administration of Ad-shOXCT1 markedly exacerbated skin fibrosis as characterized by thickening of the dermis and deposition of disorganized collagen bundles in BLM-treated mice. In accordance with *in vitro* observations, we found that introduction of Ad-shOXCT1 alone induced a spontaneous skin fibrosis (Fig. 3, *I*–*K*). The enhancement in skin fibrogenesis was further revealed by increased expression of *Col1a1*, *Col3a1*, *Acta1*, and *Fn1* (Fig. 3*M*), along with proinflammatory cytokines, such as IL-6, IL-1β, and tumor necrosis factor-α (Fig. 3*L*), in BLM-treated skin. More importantly, with reduction in the number of OXCT1-positive cells in ad-shOXCT1-administrated skin, COL1A1 relative fluorescent intensity was significantly increased as determined by immunofluorescence analysis (Fig. 3, *N*–*P*).

Taken together, these results demonstrate that dysfunction of ketone body metabolism induced by OXCT1 suppression induced a spontaneous onset of skin fibrogenesis and exacerbated BLM-induced fibrotic skin in mice.

### AcAc administration ameliorated skin fibrosis

**Figure 4:**
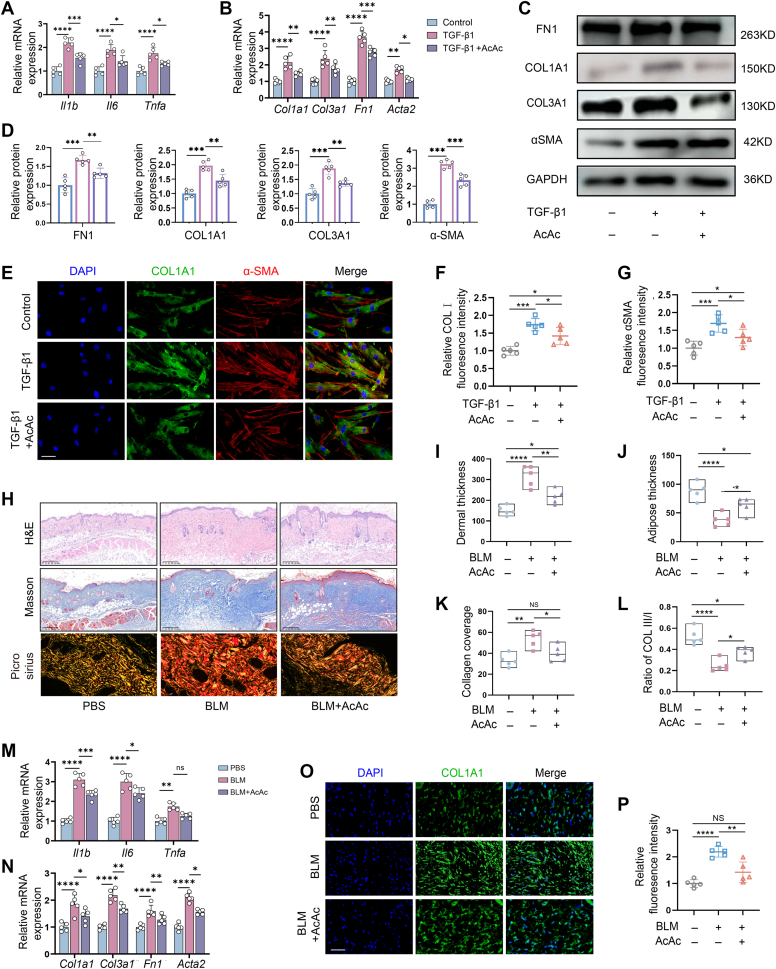
**AcAc administration attenuated fibrogenesis in bleomycin (BLM)-treated skin.***A* and *B*, qRT–PCR of inflammation- (*Il6*, *Il1b*, and *Tnfa*) and fibrosis-associated genes (*Col1a1*, *Col3a1*, *Acta1*, and *Fn1*) in AcAc-treated dermal fibroblasts in the presence of TGF-β1, with β-actin as the internal control. (n = 5 biological replicates per group). *C* and *D*, Western blot of α-SMA, COL1A1, COL3A1, and FN1 in TGF-β1-treated dermal fibroblasts with or without AcAc (50 μM)for 24 h. GAPDH served as a loading control. Semiquantitative analysis of Western blot for FN1, COL1A1, COL3A1, and αSMA. *E*, representing images of costaining of α-SMA (*red*) and COL1A1 (*green*) in dermal fibroblasts. The scale bar represents 50 μm. *F* and *G*, quantitative analysis of mean fluorescence intensity of α-SMA and COL1A1. (n = 5 biological replicates per group). *H*, H&E, Masson’s trichrome, and Sirius red staining of BLM-treated skin with or without administration of AcAc (3 mg/kg). The scale bar represents 200 μm. *I*–*L,* quantitative analysis of dermal (*I*) and adipose thicknesses (*J*), collagen coverage (*K*), and the ratio of type III/I collagen (*L*) in skin using ImageJ. (n = 5 biological replicates per group). *M* and *N*, real-time RT–PCR of inflammation- (*Il6*, *Il1b*, and *Tnfa*) and fibrosis-associated genes (*Col1a1*, *Col3a1*, *Acta1*, and *Fn1*) in BLM-treated skin with or without AcAc. (n = 5 biological replicates per group). *O*, immunofluorescent images of COL1A1 in BLM-treated skin with or without AcAc. The scale bar represents 50 μm. *P*, quantitative analysis of mean fluorescence intensity of COL1A1. (n = 5 biological replicates per group). Data expressed as the mean ± SEM. Statistical significance was analyzed using one-way analysis of variance with multiple comparisons. ∗*p* < 0.05, ∗∗*p* < 0.01, ∗∗∗*p* < 0.001, and ∗∗∗∗*p* < 0.0001. AcAc, acetoacetate; FN1, fibronectin 1; qRT–PCR, quantitative RT–PCR; TGF-β1, transforming growth factor-beta.

We next investigated whether remodeling ketone metabolism would rescue the pathology of skin fibrosis by administration of AcAc in TGF-β1-treated human dermal fibroblasts. We found that, with the addition of AcAc, expression levels of fibrogenesis-associated markers, including COL1A1, COL3A1, fibronectin, and α-SMA, were markedly reduced in TGF-β1-treated fibroblasts, which was determined by qRT–PCR (Fig. 4*A*) and Western blot analysis (Fig. 4, *C* and *D*), respectively. Meanwhile, expression of proinflammatory cytokines induced by TGF-β1, including IL6, IL1-β, and tumor necrosis factor-α, was significantly reduced by exogenous AcAc administration (Fig. 4*B*). To further evaluate the inhibitory effect of AcAc on myofibroblast activation, we assessed additional key cellular behaviors, including proliferation and migration. Cell Counting Kit-8 assays revealed that AcAc significantly attenuated the hyperproliferation of dermal fibroblasts induced by TGF-β1 (Fig. S4.1*A*). Scratch wound assays further demonstrated that AcAc inhibited fibroblast migration in a dose-dependent manner, with 50 μM AcAc significantly reducing wound closure rates over 48 h compared with TGF-β1-treated controls (Fig. S4.1, *B* and *C*). Double fluorescent staining of COL1A1 and α-SMA exhibited that AcAc administration significantly decreased expression of COL1A1 (*p* < 0.05) and α-SMA (*p* < 0.05) in TGF-β1-treated fibroblasts (Fig. 4, *E*–*G*).

To address whether topical administration of AcAc would ameliorate skin fibrosis *in vivo*, we performed subcutaneous injection of AcAc (3 mg/kg) together with BLM (5 mg/kg) in mice every other day for 4 weeks. At 4 weeks postinjection, the skin that received AcAc injection exhibited remarkable improvement in fibrogenesis induced by the BLM injection. Histological observation showed that AcAc efficiently improved skin fibrosis (Fig. 4*H*), which was further quantitatively determined by reduction in thickening of dermis (Fig. 4*I*), atrophy of hypodermal fat (Fig. 4*J*), and increase in collagenous matrix (Fig. 4*K*) and collagen type III/I ratio (Fig. 4*L*). Moreover, with administration of AcAc, the elevated mRNA levels of fibrotic- and inflammatory-associated markers induced by BLM were significantly decreased (Fig. 4, *M* and *N*). Meanwhile, accumulation of COL1A1 was significantly decreased by AcAc as detected by immunofluorescent staining (Fig. 4, *O* and *P*). Collectively, these results suggest that remodeling of ketone metabolism by AcAc supplementation prevented the development of BLM-induced skin fibrosis.

To evaluate whether AcAc could reverse established skin fibrosis under a therapeutic intervention regimen, we conducted an additional experiment in which AcAc administration was initiated after 2 weeks of BLM injection. Mice were subcutaneously injected with BLM (5 mg/kg) every other day for 2 weeks to induce fibrosis, followed by coadministration of AcAc (3 mg/kg) with BLM for another 2 weeks. AcAc treatment significantly ameliorated BLM-induced fibrotic changes, including reduced dermal thickness (456.3 μm *versus* 392.6 μm), attenuated hypodermal adipose layer atrophy (48.8 μm *versus* 78.4 μm), decreased collagen deposition (52.5% *versus* 43.8%), and a restored collagen III/I ratio (0.34 *versus* 0.42) (Fig. S1) (Fig. S4.4). Consistently, AcAc downregulated the mRNA expression of fibrotic (Col1a1, Col3a1, Acta2, and Fn1) and inflammatory (Il6, Il1b, and Tnfα) markers (Fig. 4, *M* and *N*) and reduced COL1A1 accumulation according to immunofluorescence staining (Fig. 4, *O* and *P*). These results demonstrate that therapeutic AcAc administration effectively reverses pre-existing skin fibrosis.

**Figure 5:**
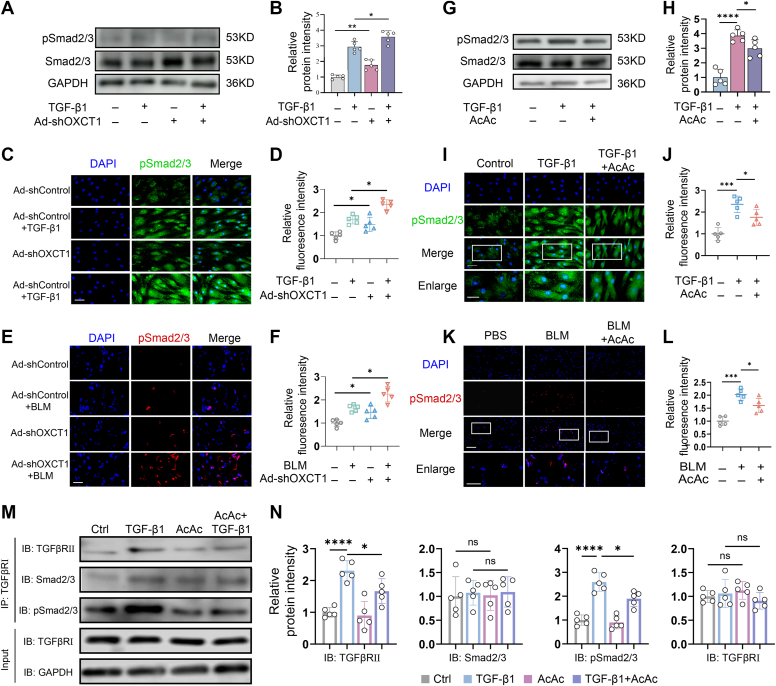
**AcAc inhibited activation of the Smad2/3 signaling pathway.***A* and *B*, Western blot (*left*) and relative intensity analysis (*right*) of phosphorylated Smad2/3 in TGF-β1-induced dermal fibroblasts with or without Ad-shOXCT1 transfection. Tubulin was blotted as a loading control (*n* = 5 biological replicates per group). *C* and *D*, immunofluorescent staining (*left*) and mean fluorescence intensity (*right*) of p-Smad2/3 in TGF-β1-induced dermal fibroblasts with transfection of Ad-shOXCT1 or controls (the scale bar represents 50 μm). The *lowest panel* is an enlarged inset of the *upper images* (the scale bar represents 200 μm). *E* and *F*, immunofluorescent images (*left*) and mean fluorescence intensity (*right*) of p-Smad2/3 in bleomycin-induced skin with Ad-shOXCT1 or Ad-shControl (the scale bar represents 50 μm). *G* and *H*, Western blot of p-Smad2/3 (*left*) and relative intensity (*right*) in TGF-β1-treated dermal fibroblasts with or without AcAc (50 μM) for 24 h. GAPDH was blotted as a loading control (*n* = 5 biological replicates per group). *I* and *J*, immunofluorescent staining (*left*) and mean fluorescence intensity (*right*) of pSmad2/3 in TGF-β1-treated dermal fibroblasts with or without AcAc. The scale bar represents 50 μm, the lowest images are enlarged insets of the *upper panel* (the scale bar represents 200 μm) (*n* = 5 biological replicates per group). K and *L*, immunofluorescent images (*left*) and mean fluorescence intensity (*right*) of p-Smad2/3 in bleomycin-induced skin with or without AcAc administration (the scale bar represents 50 μm). The lowest images are enlarged insets of the *upper panel* (the scale bar represents 200 μm). *M*, cultured fibroblast lysates are immunoprecipitated with anti-TGFβRI, followed by immunoblotting using antibodies against TGFβRII, p-Smad2/3, Smad2/3, and TGFβRI. *N*, TGFβRⅠ–TGFβRⅠ, TGFβRⅠ–Smad2/3, and TGFβRⅠ–pSmad2/3 densitometric values were normalized to TGFβRⅠ protein level in input using ImageJ. Data expressed as the mean ± SEM. Statistical significance was analyzed using one-way analysis of variance and two-way analysis of variance with multiple comparisons. ∗*p* < 0.05, ∗∗*p* < 0.01, ∗∗∗*p* < 0.001, and ∗∗∗∗*p* < 0.0001. AcAc, acetoacetate; TGF-β1, transforming growth factor beta 1.

We also examined the effect of systemic ketosis induced by a ketogenic diet (KD) on BLM-induced skin fibrosis. After 28 days of KD feeding, circulating β-OHB levels were significantly elevated (2.94 mM), whereas AcAc concentration remained unchanged (0.74 mM *versus* 0.82 mM). KD feeding did not alter skin architecture in normal mice and failed to attenuate BLM-induced dermal thickening, adipose layer atrophy, or collagen deposition (Fig. S2) (Fig. S4.2). These results suggest that elevated systemic ketone levels, particularly β-OHB, do not influence skin fibrosis, indicating that the antifibrotic effects of AcAc are likely mediated through direct actions on dermal fibroblasts rather than indirect systemic mechanisms. This interpretation is supported by our *in vitro* data showing AcAc suppresses TGF-β1-induced myofibroblast transition (Figs. 4, *A*–*G* and 5, *G*–*J*).

### Impairment in ketone metabolism enhanced the TGF-β1–Smad2/3 pathway

As the Smad pathway plays a critical role in mediating the profibrotic effect of TGF-β1, we investigated the role of AcAc in regulating Smad2/3 signaling in fibroblasts. We found that, with knockdown of OXCT1 by Ad-shOXCT1 transfection, phosphorylation levels of Smad2/3 induced by TGF-β1 were markedly increased in human dermal fibroblasts as determined by Western blot (Fig. 5, *A* and *B*). In accordance with this, immunofluorescent staining showed that fluorescent intensity of activated Smad2/3 in Ad-shOXCT1-transfected cells was significantly higher than that in cells treated with TGF-β1 alone (Fig. 5, *C* and *D*). These results indicated that impairment in ketone body metabolism largely affected activation of the TGF-β1–Smad2/3 pathway, we thus examined whether knockdown of OXCT1 would lead to an enhancement of TGF-β1–Smad2/3 activation in BLM-treated skin, where increased activation of TGF-β1–Smad2/3 has been observed. By subcutaneously injecting Ad-shOXCT1 in BLM-treated skin, we were able to find that the phosphorylation of Smad2/3 was significantly increased (Fig. 5, *E* and *F*).

Given that AcAc treatment ameliorates skin fibrosis and that dysregulation of ketone metabolism resulted in an increase in TGF-β1–Smad2/3 activation, we explored whether the therapeutic effect of AcAc on skin fibrosis is mediated by inhibiting the TGF-β1–Smad2/3 pathway in human fibroblasts. As shown in Western blot analysis, TGF-β1 induced remarkable upregulation of Smad2/3 phosphorylation in human dermal fibroblasts. With the addition of AcAc, phosphorylation of Smad2/3 was significantly suppressed (Fig. 5, *G* and *H*, *p* < 0.05). By immunostaining analysis, the suppression of nuclear translocation of pSmad2/3 by AcAc in TGF-β1-stimulated fibroblasts was observed (Fig. 5, *I* and *J*). *In vivo*, subcutaneous administration of AcAc over 4 weeks resulted in significant downregulation of p-Smad2/3 in BLM-induced fibrotic skin (Fig. 5, *K* and *L*, *p* < 0.05). Thus, these results suggest that the effect of AcAc in rescuing skin fibrosis is, at least in part, mediated through suppressing the TGF-β1–Smad2/3 signaling.

In order to further investigate the internal interaction in the TGF-β–Smad signaling pathway after AcAc treatment, coimmunoprecipitation was carried out in fibroblasts. The combination of TGFβRII and TGFβRI was more in TGF-β1-treated cells than in normal conditions (Fig. 5, *M* and *N*). This complex then binds to and phosphorylates Smad2/3. The probability of the p-Smad2/3 combination with TGFβRII and TGFβRI was enhanced, and the ratio of p-Smad2/3/Smad2/3 was increased after TGF-β1 exposure. AcAc treatment significantly inhibited the interaction of TGFβRI with TGFβRII, p-Smad2, and p-Smad3 but did not affect the interaction of TGFβRI with Smad2/3. AcAc could weaken the combination between TGF receptors and p-Smad2/3, inhibit the phosphorylation of Smad2/3 to suppress activation of the TGF-β–Smad signaling pathway by direct or indirect stimulation, but not affect total Smad2/3. These results indicated that AcAc inhibited Smad2/3 phosphorylation by selectively blocking Smad2/3–TGFβRI interaction.

## Discussion

**Figure 6:**
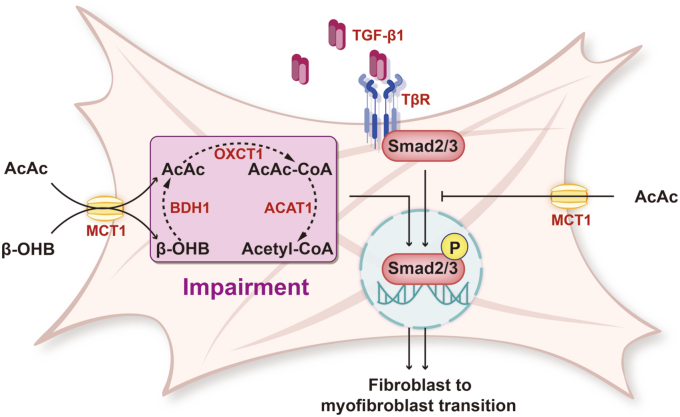
**Schematic diagram depicting the mechanism by which OXCT1-mediated AcAc metabolism ameliorates skin fibrosis.** Disorders in ketone body metabolism can induce or promote the transition of fibroblasts to myofibroblasts. This occurs through the enhanced phosphorylation of Smad2/3, leading to extracellular matrix deposition and skin fibrosis. Exogenous AcAc can inhibit TGF-β1-induced phosphorylation of Smad2/3 to counteract this process. AcAc, acetoacetate; OXCT1, 3-oxoacid CoA-transferase 1; TGF-β1, transforming growth factor beta 1.

Skin fibrosis, characterized by excessive proliferation of skin fibroblasts and deposition of ECM, is the histopathologic hallmark of many dermatologic diseases, such as scleroderma, HSs, and keloids (15, 16). Despite numerous studies on the pathological mechanism of skin fibrosis, there is a scarcity of interventions and treatments that have progressed to clinical trials (17). In the present study, our findings revealed a reduction in the expression of BDH1 and OXCT1 in settings of skin fibrosis, including HS and BLM-induced mouse skin. We found that remodeling ketone body metabolism by AcAc supplementation remarkably ameliorated skin fibrosis in mouse skin treated with BLM (Fig. 6).

Fibrogenesis has been defined as a metabolic disease, which involves a complex network of metabolic pathways that orchestrates fundamental cellular behaviors (18, 19, 20). For example, diabetic cardiomyopathy largely relies on ketone bodies as an alternative energy source (21). Reduction in activity of BDH1 and OXCT1 leads to exacerbation of myocardial fibrosis resulting from oxidative damage. In skin fibrosis, metabolic dysregulations have been associated with abnormal cell proliferation, differentiation, and excessive ECM deposition. Zhao *et al.* (22) observed the reciprocal role of downregulation in fatty acid oxidation and upregulation in glycolysis in the progression of skin fibrogenesis, respectively, underscoring the multifaceted outcomes of metabolic remodeling in regulating ECM homeostasis in skin. Keloids represent a typical model for skin fibrosis, which is characterized by progressive accumulation of dense collagenous matrix that is usually beyond the original wound (16). By analyzing public RNA-Seq data of keloid-derived fibroblasts, we were able to identify a significant fold change in expression of OXCT1 and BDH1 in keloid-derived fibroblasts, which was consistently manifested in the whole transcriptome data for BLM-treated skin in mice. The impairment of ketone body metabolism, as characterized by decreased expression of OXCT1 and BDH1, was also detected in human HS- and BLM-induced fibrotic skin. Thus, the dysfunction of ketone body metabolism highlights their potential role in regulating skin fibrosis.

In the context of starvation or carbohydrate restriction, ketone bodies, mainly β-OHB and AcAc, serve as alternative fuels for the mitochondria in extrahepatic cells, producing two molecules of AcCoA and one molecule of succinate, both of which subsequently enter the tricarboxylic acid cycle (10). The effects of ketogenic supplement therapy on fibrogenesis seem to vary across different organs. For instance, by enhancing ketolysis *in vivo* with either βOHB supplementation or KD feeding, fibrosis in the kidneys was markedly reversed in C57 BKS db/db mice (23). While a high-fat KD can lead to cholesterol accumulation in the liver and thus exacerbate liver fibrosis induced by CCl4 in mice (24). This discrepancy has been ascribed to the different impacts of β-OHB and AcAc on the development and progression of fibrogenesis, respectively. Given that under the circumstance of pathological disease, expression of BDH1, which is responsible for the conversion of β-OHB into AcAc, has been widely reported to be inhibited (25). Thus, AcAc was administered to replenish ketone body metabolism in this study in an attempt to impede the progression of skin fibrosis. Our results showed that inhibition of ketone body metabolism by Ad-shOXCT1 transfection significantly upregulated fibrosis-associated markers in dermal fibroblasts. Consistently, the knockdown of OXCT1 by adenoviral injection *in vivo* can induce simultaneously skin fibrogenesis and aggravated fibrosis in skin treated with BLM. More importantly, we observed a downregulated response of fibroblasts to TGF-β1 in fibrotic marker expression by exogenous supplementation of AcAc. The *in vivo* study further manifested the therapeutic effect of AcAc on skin fibrosis, as evidenced by improvement in dermal thickening and subcutaneous fat layer atrophy in BLM-treated skin. In addition, collagenous matrix deposition was significantly ameliorated with administration of AcAc. However, we acknowledge that the adenoviral delivery of shOXCT1 lacks cell-type specificity. To partially address this concern, we performed additional costaining for OXCT1 and α-SMA (Fig. S3), which supported the association between OXCT1 knockdown and myofibroblast activation. Nevertheless, the absence of a myofibroblast-specific knockout model (*e.g.*, using Periostin-Cre) remains a limitation of this study. These findings propose a novel therapeutic approach for addressing skin fibrosis by subcutaneously administering AcAc to reprogram impaired ketone body catabolism.

The canonical TGF-β1–Smad signaling pathway, which involves the activation of Smad2 and Smad3 through phosphorylation of TGF-β1 receptor 1 (ALK5), plays a central role in boosting the excessive fibrogenesis of skin, leading to aberrant collagen synthesis and deposition, a higher proportion of collagen I/III, and formation of abnormally crosslinked collagen fiber bundles (26, 27). The interaction between ketone body reprogramming and activation of the TGF-β1–Smad2/3 pathway has been well documented in different organs. In the liver of diabetic mice, it was observed that the expression level of TGF-β1 was significantly decreased in response to a ketone body diet, indicating that KD-induced TGF-β1–Smad signaling suppression contributed to improvement in hepatic fibrosis (28). Intriguingly, it was shown that supplementation of β-OHB alone unexpectedly resulted in activation of the TGF-β1–Smad pathway in HK-2 cells (23). Thus, we turned to investigate the impact of AcAc on the TGF-β1–Smad pathway during the progression of skin fibrogenesis. We found that the knockdown of OXCT1 by adenovirus increased the expression of p-Smad2/3 in fibroblasts. We found that AcAc treatment significantly downregulated the phosphorylation of Smad2/3 in TGF-β1-treated fibroblasts as well as in fibrotic skin of mice. Together, these results suggested that the amelioration of skin fibrosis by AcAc was mediated, at least in part, *via* suppression of the TGF-β1–Smad pathway. However, the mechanism by which ketone bodies regulate the TGF-β1–Smad pathway remains to be explored in our future works. While our data establish TGF-β1–Smad2/3 suppression as the primary mechanism, further studies will explore potential modulation of Hippo–Yes-associated protein–TEA domain and inflammatory cascades by ketone body metabolites.

In conclusion, our study provides experimental evidence supporting the dysfunction of ketone body metabolism associated with the onset and exacerbation of skin fibrosis. Notably, subcutaneous administration of AcAc resulted in remarkable improvement in impeding the progression of skin fibrogenesis. A mechanistic study showed that the effect of AcAc on ameliorating skin fibrogenesis is mediated by suppressing the TGF-β1–Smad2/3 signaling pathway. Our findings thus indicated that administration of AcAc could serve as a potential therapeutic avenue to disrupt the pathology in skin fibrogenesis.

## Experimental procedures

### Bioinformatic analysis

Transcriptome datasets were obtained from National Center for Biotechnology Information Gene Expression Omnibus libraries GSE145725 and GSE226331, where relative gene expression levels were calculated using Cufflinks v2.2.1 in fragments per kilobase per million reads. Transcript annotation files were obtained from the Gencode v22 Comprehensive Gene Annotation gtf file (http://www.gencodegenes.org). EdgeR, a Bioconductor software package, was used to identify DEGs between normal skin tissue and fibrotic keloids for Gene Ontology pathway analysis, the Kyoto Encyclopedia of Genes and Genomes analysis, and to draw Volcano plots and heat maps. DEGs were identified using the Bioconductor package edgeR (version 3.40.2) with a threshold of |log2fold change| >1.5 and a false discovery rate–adjusted *p* < 0.05. A PPI network was created utilizing the Search Tool for Retrieval of Interacting Genes (STRING) database (https://string-db.org/), in which *Homo sapiens* was the only species considered. The PPI network was constructed by mutual mapping of disease targets and drug targets, incorporating the mapping results into the STRING database.

### Collection of HS and normal skin

This study was conducted with the approval of the Shanghai Ninth People’s Hospital for the use of discarded human tissues. All participants provided written informed consent. The research protocol adhered to relevant ethical guidelines governing human participant research and complied with the principles of the Declaration of Helsinki. Both normal and hyperplastic scar tissues were clinically and pathologically confirmed, with detailed characteristics summarized in Table S1. Specimens included hyperplastic scar tissues from five patients and normal skin tissues from five healthy controls. After collection, tissue samples were analyzed for real-time qPCR. Additional sections were prepared for histological evaluation. The remaining materials were used for primary fibroblast culture.

### Creation of skin fibrosis in mice

Fifteen 8-week-old male BALB/c mice (weighing 30–35 g) were purchased from Shanghai SLAC Laboratory Animal Co Ltd and maintained under specific pathogen-free conditions in the animal center of Tongji University School of Medicine. All procedures were approved by the Animal Research Committee of Tongji University.

As reported previously (29), skin fibrosis was induced by subcutaneously injecting BLM (5 mg/kg) into a one-square-centimeter area of dorsal skin in mice every 2 days for 4 weeks (BLM group, n = 5). Administration of AcAc was performed by subcutaneous injection of AcAc (3 mg/kg) in combination with BLM every other day for 4 weeks (AcAc group, n = 5). Injection of PBS (Invitrogen) served as a control (PBS group, n = 5). After 28 days, dorsal skin with a subcutaneous adipose layer was harvested for histology and qRT–PCR.

### Histological and immunofluorescent staining

Samples of normal human skin, HS, and BLM-treated dorsal skin of mice were fixed with formalin and then embedded with paraffin, respectively. Following serial section in 4 μm, slices were deparaffinized and dehydrated and subjected to H&E, Masson’s trichrome, and Sirius red staining. For immunofluorescent staining, mouse monoclonal anti-COL1A1 (1:500 dilution, #PA5-29569; Thermo Fisher Scientific), rabbit polyclonal anti-BDH1 (1:100 dilution, #15417-1-AP; Proteintech), rabbit polyclonal anti-OXCT1 (1:600 dilution, #12175-1-AP; Proteintech), and rabbit polyclonal anti–p-Smad2/3 (1:100 dilution, #PA5-99378; Thermo Fisher Scientific) were incubated at 4 °C overnight. Slides were washed and incubated for 1 h with Alexa Fluor 488–conjugated goat anti-mouse IgG (1:500 dilution) and Alexa Fluor 568–conjugated goat anti-rabbit IgG (1:500 dilution). Nuclei were stained for 10 min at room temperature with 5 μg/ml Hoechst 33242 from Thermo Fisher Scientific. Images were obtained using a Fluoview FV10i confocal microscope or a BX53 fluorescence microscope from Olympus. For quantification, integrated density was analyzed using ImageJ 1.x (National Institutes of Health).

### AcAc and BHB measurement

BHB and AcAc levels were measured in the serum of each mouse from all experimental groups. Blood was collected from the orbital vein into procoagulation tubes. Serum samples were obtained by centrifugation at 1000*g* for 10 min at 4 °C, followed by BHB (Elabscience; E-BC-K785-M) and AcAc (Abcam; ab180875) measurements using a colorimetric assay kit according to the manufacturer’s instructions.

### Cultivation of dermal fibroblasts

The freshly harvested dermal tissue was thoroughly washed in PBS after removing epidermal and adipose tissues and subjected to digestion with type I collagenase (Worthington) at 37 °C for 1 h. Following digestion, cell pellets were obtained by centrifuging at 400*g* for 10 min and resuspended in culture medium consisting of Dulbecco’s modified Eagle’s medium (Gibco), supplemented with 10% fetal bovine serum (Hyclone) and 1% penicillin–streptomycin. The resuspended fibroblasts were cultured at 37 °C with 5% CO_2_ for approximately 14 days. When reaching 80% to 90% confluence, cells were passaged, and those at passage 3 to 5 were used in the following study.

Transition of fibroblasts to myofibroblasts was established by treating cells with TGF-β1 (10 ng/ml) for 24 h as previously reported (30). The effect of AcAc on cell phenotype transition was observed by adding AcAc (50 μM) in culture medium in the presence or absence of TGF-β1 (10 ng/ml), respectively.

### Knockdown of OXCT1 *in vitro* and *in vivo*

Adenoviruses engineered to express mouse OXCT1 shRNA (Ad-shOXCT1) were procured from Wei Nuo Sai Biology. These adenoviruses were propagated by infecting human embryonic kidney-293a cells for a duration of 48 h. Subsequently, the human embryonic kidney cells were collected and lysed through a series of freeze–thaw cycles to release the adenoviruses, which were then subjected to CsCl density gradient ultracentrifugation for purification.

Fibroblasts at passage 1, reaching a confluence of 70 to 80%, were cultured in serum-free medium for 12 h prior to transfection with either Ad-shControl or Ad-shOXCT1 at a multiplicity of infection of 800 for 48 h, thereby establishing stable fibroblasts with OXCT1 knockdown. Followed by a 24-h culture period with or without TGF-β1 (10 ng/ml) before undergoing further analysis.

Knockdown of OXCT1 *in vivo* was performed by subcutaneous injection of Ad-shOXCT1 once a week for 4 weeks with or without BLM (Ad-shOXCT1 group, Ad-shOXCT1 + BLM group, n = 5). Subcutaneous injection of empty adenovirus (Ad-shControl) was used as a control (Ad-shControl group, Ad-shControl + BLM group, n = 5). Four weeks postinitial injection, the mice were euthanized, and their skin was meticulously harvested for subsequent analysis. Applied titer of the adenovirus was 1∗108 pfu.

### Quantitative real-time PCR

Total RNA was extracted with TRIzol (R401; Vazyme) and subsequently reverse-transcribed into complementary DNA with HiScript II Q RT SuperMix (R222-01; Vazyme) in preparation for qPCR. qRT–PCR was consequently conducted with an Applied Biosystems 7500 device with ChamQ SYBR qPCR Master Mix (Q331-02; Vazyme). 2-ΔΔCT approach was employed to calculate the relative expression levels, which were in turn normalized to β-actin. The sequences of all primers are illustrated in Table S2.

### Western blot analysis

Cells were implanted into 6-well plates and cultured until attachment. TGF-β1, homocysteine, and tetrahydrofolate were added correspondingly and incubated. The protein concentration was determined using the Pierce BCA protein assay kit (Thermo Fisher Scientific). The collected proteins were separated through SDS-PAGE and electrotransferred to polyvinylidene fluoride membranes. After blocking, the membranes were reacted with the corresponding primary antibodies against BDH1 (1:5000 dilution, 15417-1-AP; Proteintech), OXCT1 (1:6000 dilution, 12175-1-AP; Proteintech), COL1A1 (1:1000 dilution, TA7001; Abmart), COL3A1 (1:1000 dilution, T510299; Abmart), α-SMA (1:1000 dilution, T55295; Abmart), fibronectin (1:1000 dilution, T59537; Abmart), Smad2/3 (1:4000 dilution, PA5-17621; Thermo Fisher Scientific), p-Smad2/3 (1:1000 dilution, PA5-110155; Thermo Fisher Scientific), and GAPDH (1:1000 dilution, TT0004; Abmart) overnight at 4 °C. Finally, they were incubated with horseradish peroxidase–linked rabbit IgG antibody (1:3000 dilution, 7074S/7076S; Cell Signaling Technology) for 1 h. Signals were developed with SuperSignal West Dura substrate (Thermo Fisher Scientific) and imaged with a Gel Doc XR+ device (Bio-Rad Laboratories, Inc). Quantification analysis was performed with the Volume tool in ImageLab 6.0.1 (Bio-Rad), adjusted to GAPDH expression, and normalized to respective control samples to calculate fold change to control.

### Quantitative and statistical analysis

Data were expressed as the mean ± SD. To determine the statistical significance of observed differences, we utilized one-way or two-way ANOVA, followed by Tukey’s post hoc test for multiple comparisons when appropriate. For direct comparisons between two groups, we employed unpaired two-tailed Student’s *t* tests. The levels of statistical significance were set at ∗*p* < 0.05, ∗∗*p* < 0.01, ∗∗∗*p* < 0.001, and ∗∗∗∗*p* < 0.0001. All calculations were performed using Prism, version 9.0 (GraphPad Software, Inc) on a Windows 11 operating system.

## Data availability

All data generated in this study are available on reasonable request from the corresponding author.

## Ethics approval

The study was conducted according to the guidelines of the Declaration of Helsinki and approved by the Institutional Review Board (or Ethics Committee) of Shanghai Ninth People’s Hospital, Shanghai Jiao Tong University School of Medicine (protocol code: SH9H-2021-A32-1).

## Consent for publication

We declare that all the work described here has not been published before and that its publication has been approved by all coauthors.

## Supporting information

This article contains supporting information.

## Conflict of interest

The authors declare that they have no conflicts of interest with the contents of this article.
