# Supplementary material for: Acetoacetate ameliorates skin fibrosis by modulating TGF-β1–Smad2/3 signaling pathway
Source: J Biol Chem. 2025 Oct 28;301(12):110867. doi: 10.1016/j.jbc.2025.110867 (PMC12666854; doi:10.1016/j.jbc.2025.110867)
Supplement: Supplementary Table 1 [file mmc1.docx]

Supplementary Table 1. Clinical characteristics of HS patients.

| Sex | Age | Diagnosis | Sample | Site of biopsy |
| --- | --- | --- | --- | --- |
| Female | 33y | HS | HS | Back |
| Female | 49y | HS | HS | Back |
| Female | 29y | HS | HS | Back |
| Male | 34y | HS | HS | Chest |
| Female | 33y | HS | HS | Chest |
| Male | 56y | - | Healthy | Back |
| Female | 44y | - | Healthy | Back |
| Female | 35y | - | Healthy | Back |
| Male | 39y | - | Healthy | Chest |
| Male | 31y | - | Healthy | Chest |
