# Supplementary material for: Acetoacetate ameliorates skin fibrosis by modulating TGF-β1–Smad2/3 signaling pathway
Source: J Biol Chem. 2025 Oct 28;301(12):110867. doi: 10.1016/j.jbc.2025.110867 (PMC12666854; doi:10.1016/j.jbc.2025.110867)
Supplement: Supplementary Table 2 [file mmc2.docx]

Supplementary Table 2. Primers used in this study.

| **Type** | **Gene** | **Forward Primer** | **Reverse Primer** |
| --- | --- | --- | --- |
| Mouse | GAPDH | 5′ATATCGCTGCGCTGGTCGTC3′ | 5′AGGATGGCGTGAGGGAGAGC3′ |
|  | Acta2 | 5′GTCCCAGACATCAGGGAGTAA3′ | 5′TCGGATACTTCAGCGTCAGGA3′ |
|  | Col1a1 | 5′GCTCCTCTTAGGGGCCACT3′ | 5′CCACGTCTCACCATTGGGG3′ |
|  | Col3a1 | 5′CTGTAACATGGAAACTGGGGAAA3′ | 5′CCATAGCTGAACTGAAAACCACC3′ |
|  | Fn1 | 5′ATGTGGACCCCTCCTGATAGT3′ | 5′GCCCAGTGATTTCAGCAAAGG3′ |
|  | Bdh1 | 5′CCACCTGCAAGACCATCGAC3′ | 5′CTGGCGAGCCTTAGTITGGAC3′ |
|  | Oxct1 | 5′CCAGGCACCTGTGGTCAAC3′ | 5′GGTCTCGTGATTGGATCTGCT3′ |
|  | Acat1 | 5′CTGGGCACTGTTATCCATCCC3′ | 5′TCCTGCTGATAGAGGGTGGC3′ |
|  | Mct1 | 5′ATGATCCAGCGGTACAAACTAAG3′ | 5′CATCCGGTAGGCCAAGTGTTC3′ |
|  | Il-1β | 5′TGCCACCTTTIGACAGTGATG3′ | 5′TGATGTGCTGCTGCGAGATT3′ |
|  | Il-6 | 5′AGCCAGAGTCCTTCAGAGAGAT3′ | 5′AGAGCATIGGAAATTGGGGT3′ |
|  | Tnf | 5′CCCTCACACTCAGATCATCTTCT3′ | 5′GCTACGACGTGGGCTACAG3′ |
|  | Ccl2 | 5′TTAAAAACCTGGATCGGAACCAA3′ | 5′GCATTAGCTTCAGATTTACGGGT3′ |
|  | Ccl5 | 5′GCTGCTTTGCCTACCTCTCC3′ | 5′TCGAGTGACAAACACGACTGC3′ |
|  | Cx3cl1 | 5′ACGAAATGCGAAATCATGTGC3′ | 5′CTGTGTCGTCTCCAGGACAA3′ |
|  | Cxcl16 | 5′CCTTGTCTCTTGCGTTCTTCC3′ | 5′TCCAAAGTACCCTGCGGTATC3′ |
| Human | β-Actin | 5′CATGTACGTTGCTATCCAGGC3′ | 5′CTCCTTAATGTCACGCACGAT3′ |
|  | Acta2 | 5′AAAAGACAGCTACGTGGGTGA3′ | 5′GCCATGTTCTATCGGGTACTTC3′ |
|  | Col1a1 | 5′GAGGGCCAAGACGAAGACATC3′ | 5′CAGATCACGTCATCGCACAAC3′ |
|  | Col3a1 | 5′GGAGCTGGCTACTTCTCGC3′ | 5′GGGAACATCCTCCTTCAACAG3′ |
|  | Fn1 | 5′CGGTGGCTGTCAGTCAAAG3′ | 5′AAACCTCGGCTTCCTCCATAA3′ |
|  | Col1a1 | 5′GGCCAAGTGTGAGTTCTTCAA3′ | 5′GGCTCGATAATCGTGTCCCC3′ |
|  | Bdh1 | 5′GAGCGGCATGAGAGACTCC3′ | 5′CCGGTCAAACCTTGAGATGAG3′ |
|  | Oxct1 | 5′AGCGGGAGAAGCTAAACGAAG3′ | 5′CGGTAGGCCAAGTGTTCAAGG3′ |
|  | Acat1 | 5′ACAGCCCGCAAGTTTGTTAAG3′ | 5′CCAGTAACCCATACCGCAGG3′ |
|  | Mct1 | 5′TCATGTTCACATCGGAGTCTGT3′ | 5′CATGCCGGTCTTGCACACT3′ |
|  | Il-1β | 5′ATGATGGCTTATTACAGTGGCAA3′ | 5′GTCGGAGATTCGTAGCTGGA3′ |
|  | Il-6 | 5′ACTCACCTCTTCAGAACGAATTG3′ | 5′CCATCTTTGGAAGGTTCAGGTTG3′ |
|  | TNF-α | 5′CCTCTCTCTAATCAGCCCTCTG3′ | 5′GAGGACCTGGGAGTAGATGAG3′ |
|  | Ccl2 | 5′CAGCCAGATGCAATCAATGCC3′ | 5′TGGAATCCTGAACCCACTTCT3′ |
|  | Ccl5 | 5′CCAGCAGTCGTCTTTGTCAC3′ | 5′CTCTGGGTTGGCACACACTT3′ |
|  | Cx3cl1 | 5′ACCACGGTGTGACGAAATG3′ | 5′TGTTGATAGTGGATGAGCAAAGC3′ |
|  | Cxcl16 | 5′CCCGCCATCGGTTCAGTTC3′ | 5′CCCCGAGTAAGCATGTCCAC3′ |
|  |  |  |  |
|  |  |  |  |
|  |  |  |  |
|  |  |  |  |
