# Supplementary material for: Acetoacetate ameliorates skin fibrosis by modulating TGF-β1–Smad2/3 signaling pathway
Source: J Biol Chem. 2025 Oct 28;301(12):110867. doi: 10.1016/j.jbc.2025.110867 (PMC12666854; doi:10.1016/j.jbc.2025.110867)
Supplement: Supplementary Figures [file mmc3.docx]

**Supplementary figures**


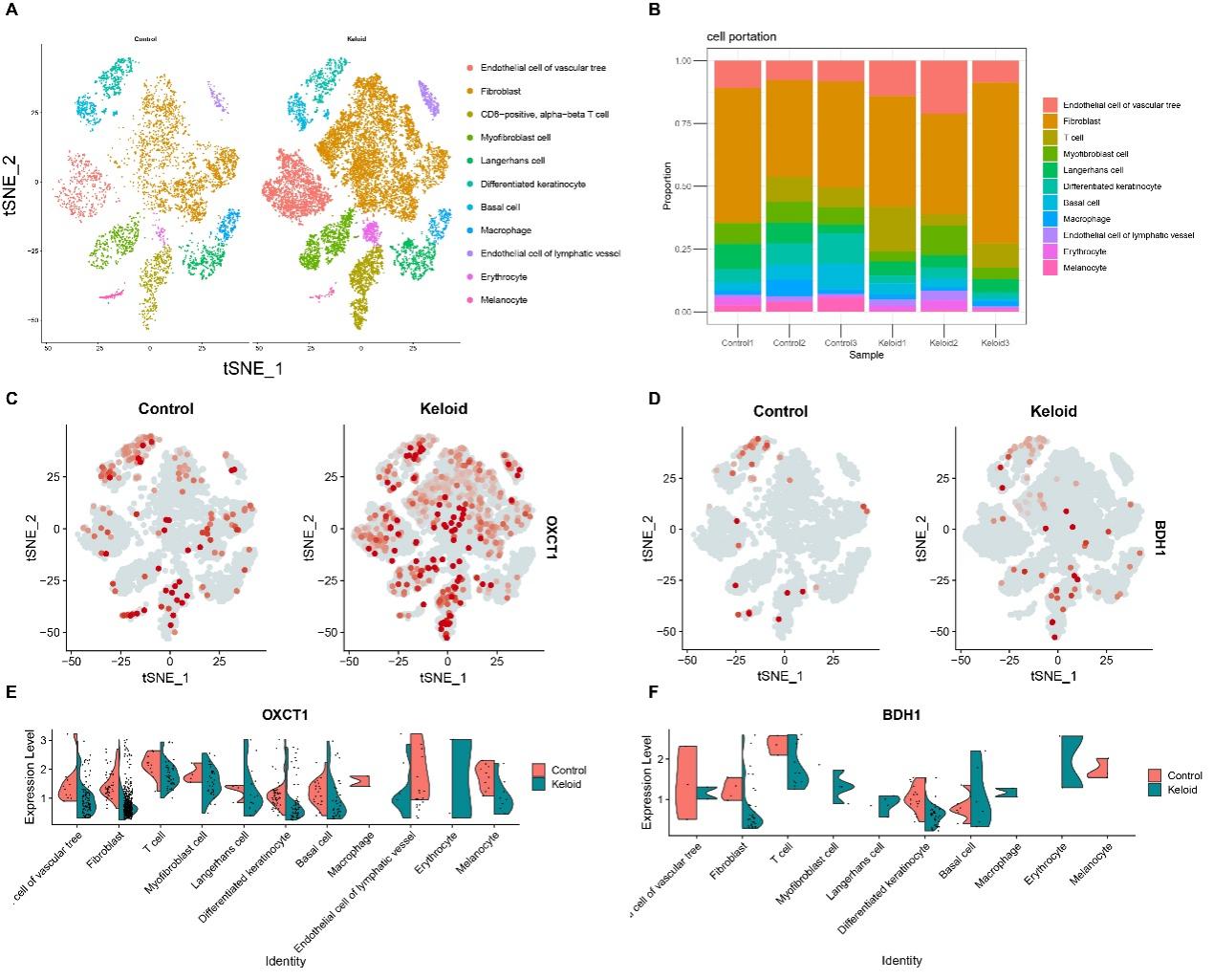


**Figure S1.1. Characterization of human skin and keloid samples through scRNA-seq in GSE220300.**

(A) Keloids and normal skin samples revealed 11 cell clusters: Endothelial cell of vascular tree, Fibroblasts, T cells, Myofibroblast, Langerhans cells, Differentiated keratinocyte, Basal cells, Macrophage, Endothelial cell of lymphatic vessel, Erythrocyte, Melanocytes. (B) The distribution of cell lineages in keloids and normal skin. (E, F) Feature plots and Violin plots show the gene of OXCT1. (G, H) Violin plots and feature plots of BDH1 in keloids and Normal skin tissue.


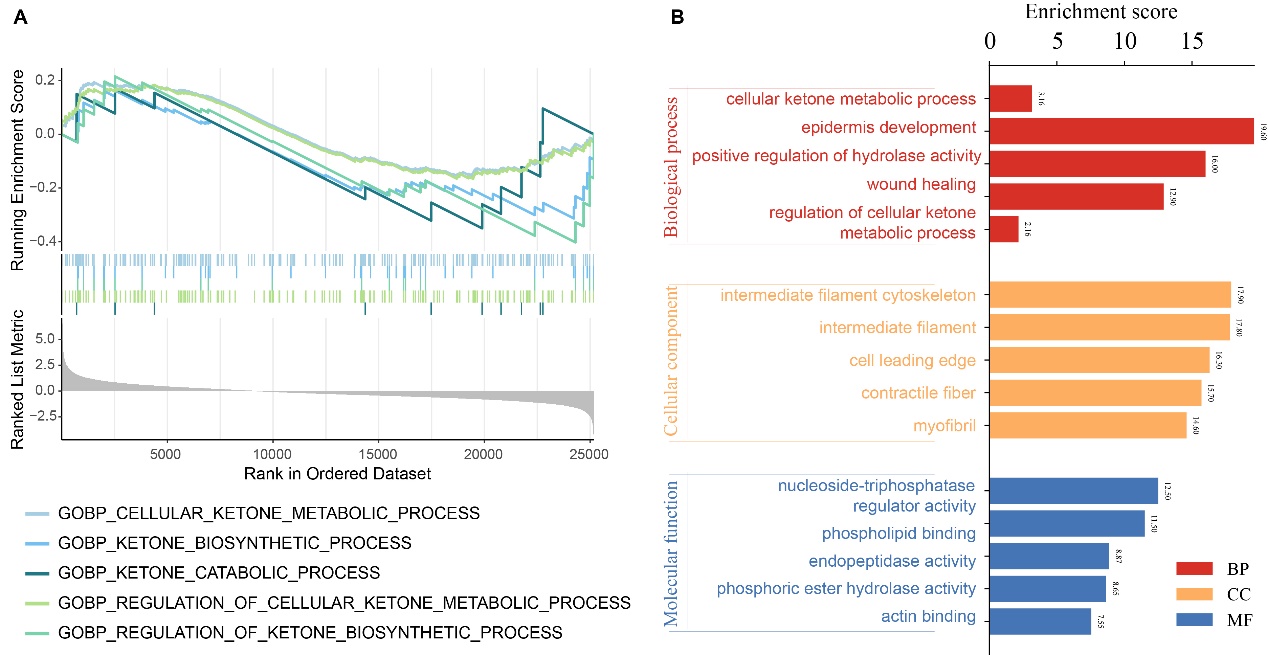


**Fig. S1.2 Gene set and functional enrichment analysis of differentially expressed genes in bleomycin-induced murine skin fibrosis.**

(A) Gene set enrichment analysis (GSEA) of response to ketones, and cellular ketone metabolic process from dataset GSE226331 . (B) GO annotated statistics based on biological process (BP), cellular component (CC), and molecular function (MF) of DEGs between keloid and healthy skin.


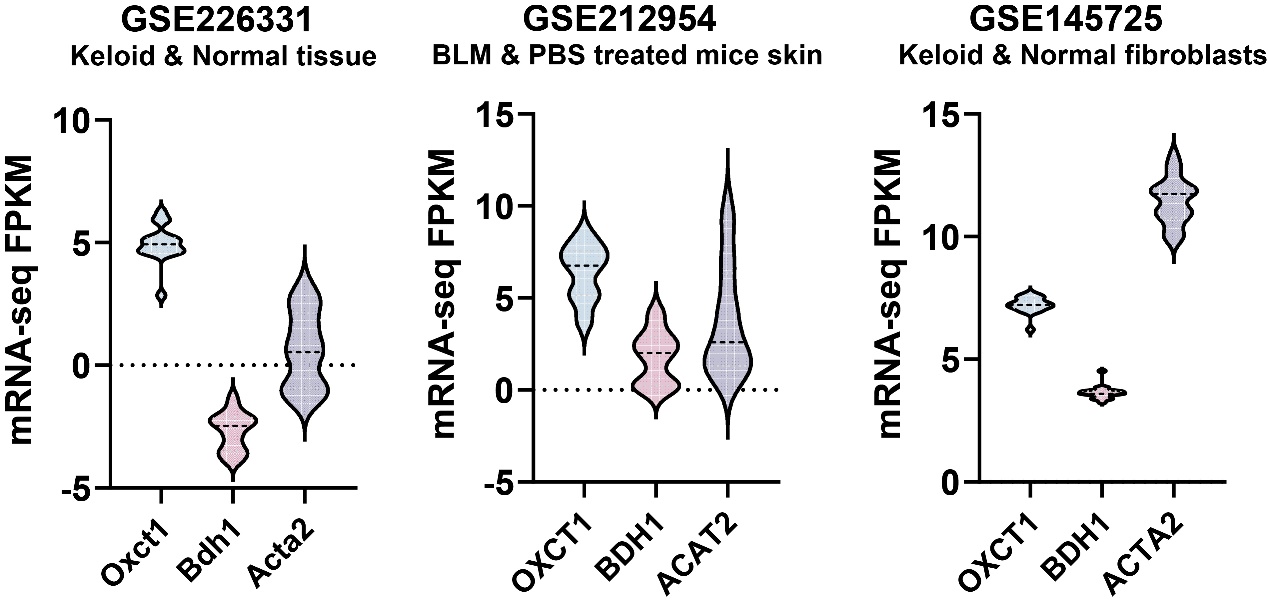


**Fig. S2. Count and FPKM values for *CBS*, *MTR*, *ACTA2* in keloid and normal skin tissue in publicly available bulk RNA-seq datasets.**


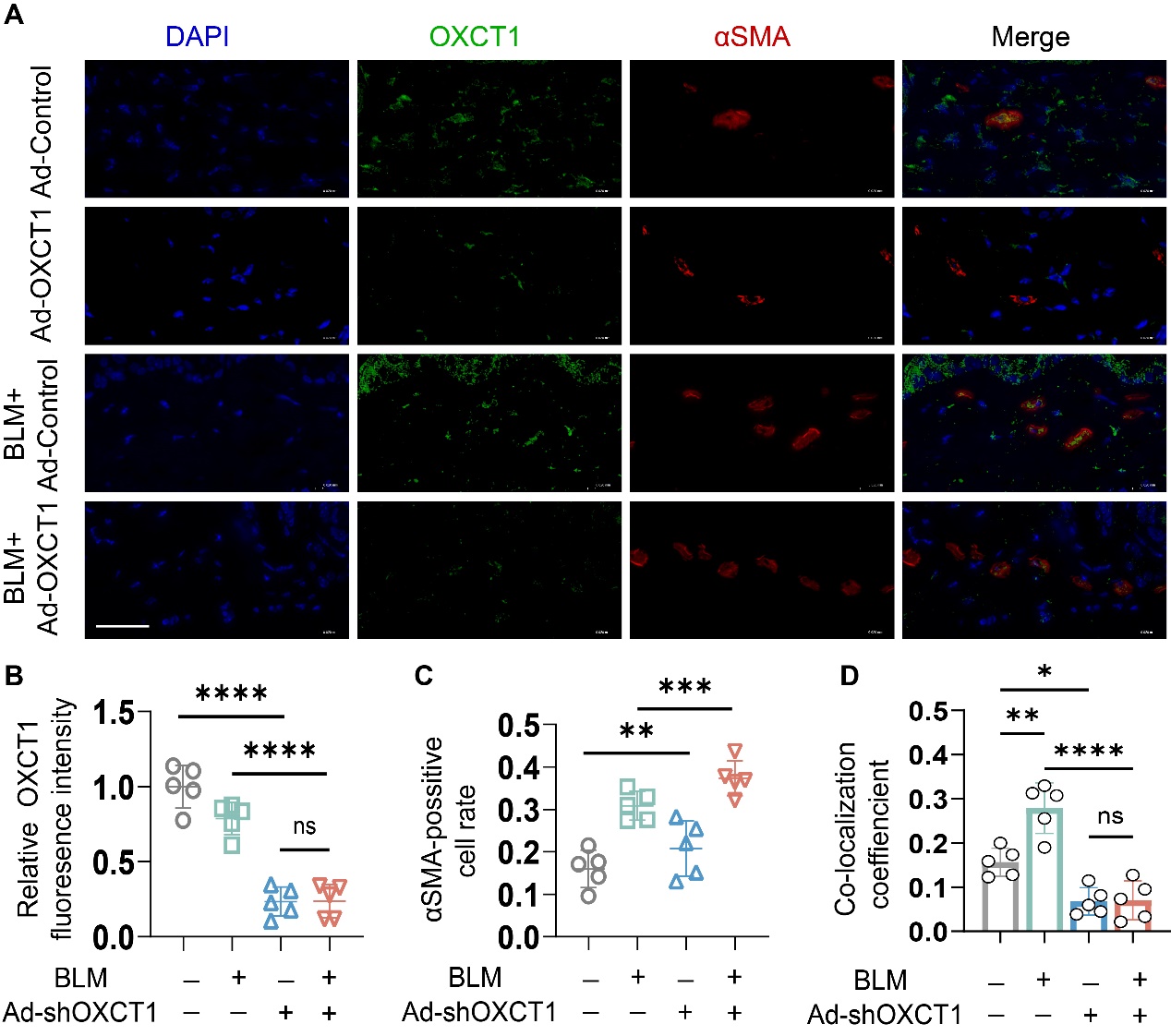


**Fig. S3 The knockout of OXCT1 induced and exacerbated the fibrotic phenotype of BLM-induced Skin fibrosis model.**

(A) Immunofluorescence images of OXCT1(green) and COL1A1red) in BLM challenged skin. Scale bar, 30 μm. (B-C) Quantitative analysis of mean fluorescence intensity of OXCT1 αSMA-possitive cell rate and quantification of co-localization between OXCT1 and αSMA. Statistical significance was analyzed using one-way analysis of variance and two-way analysis of variance with multiple comparisons. * *p* < 0.05, ** *p* < 0.01, *** *p* < 0.001, **** *p* < 0.0001.


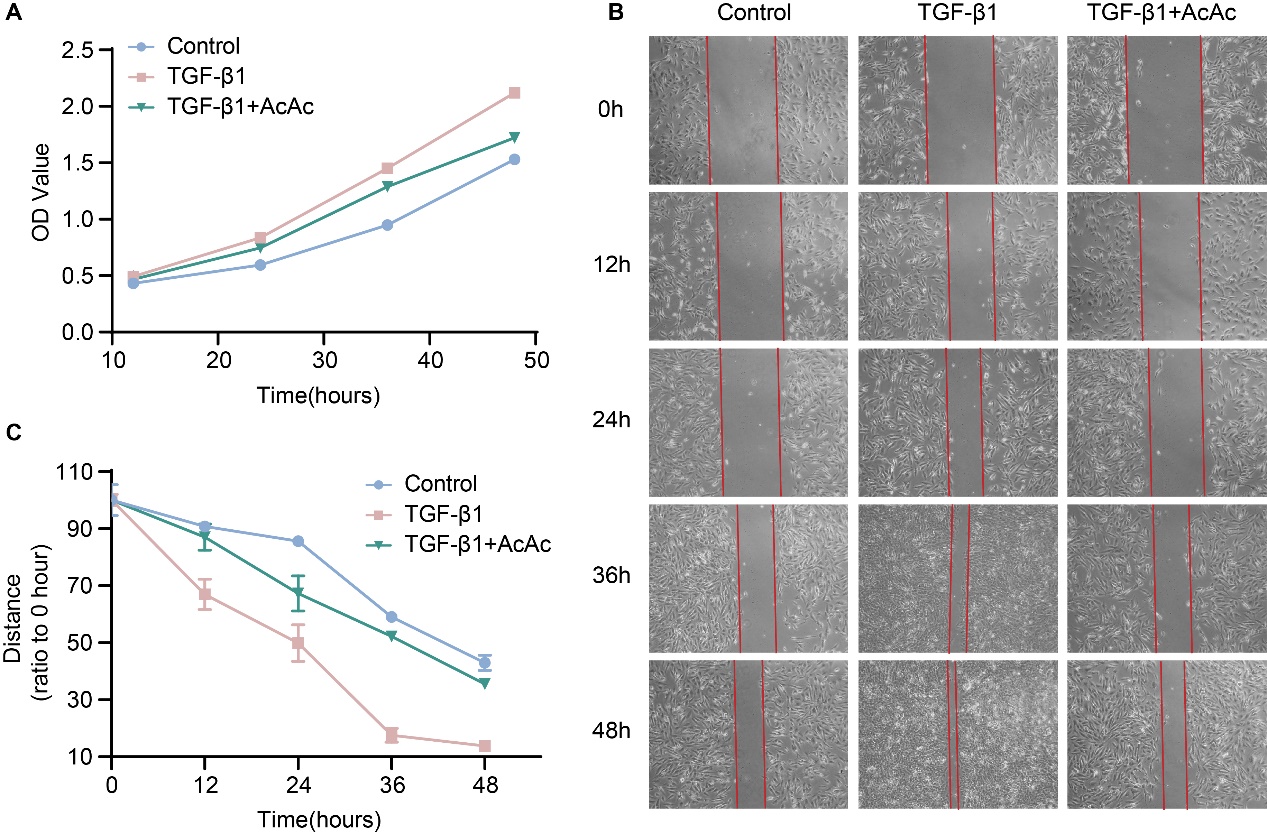


**Fig. S4.1 AcAc inhibits TGF-β1-induced fibroblast proliferation and migration.**

(A)CCK-8 assay of fibroblast proliferation under TGF-β1 ± AcAc (50µM) treatment at 12, 24, 36, and 48 h. (B-C) Wound healing assay of fibroblasts treated with TGF-β1 (10 ng/mL) ±AcAc (50µM). Representative images (B) and quantified migration rates (C) at 0, 12, 24, 36, and 48h post-scratch (scale bar, 50µm; n=3). Statistical significance was analyzed using one-way analysis of variance and two-way analysis of variance with multiple comparisons.


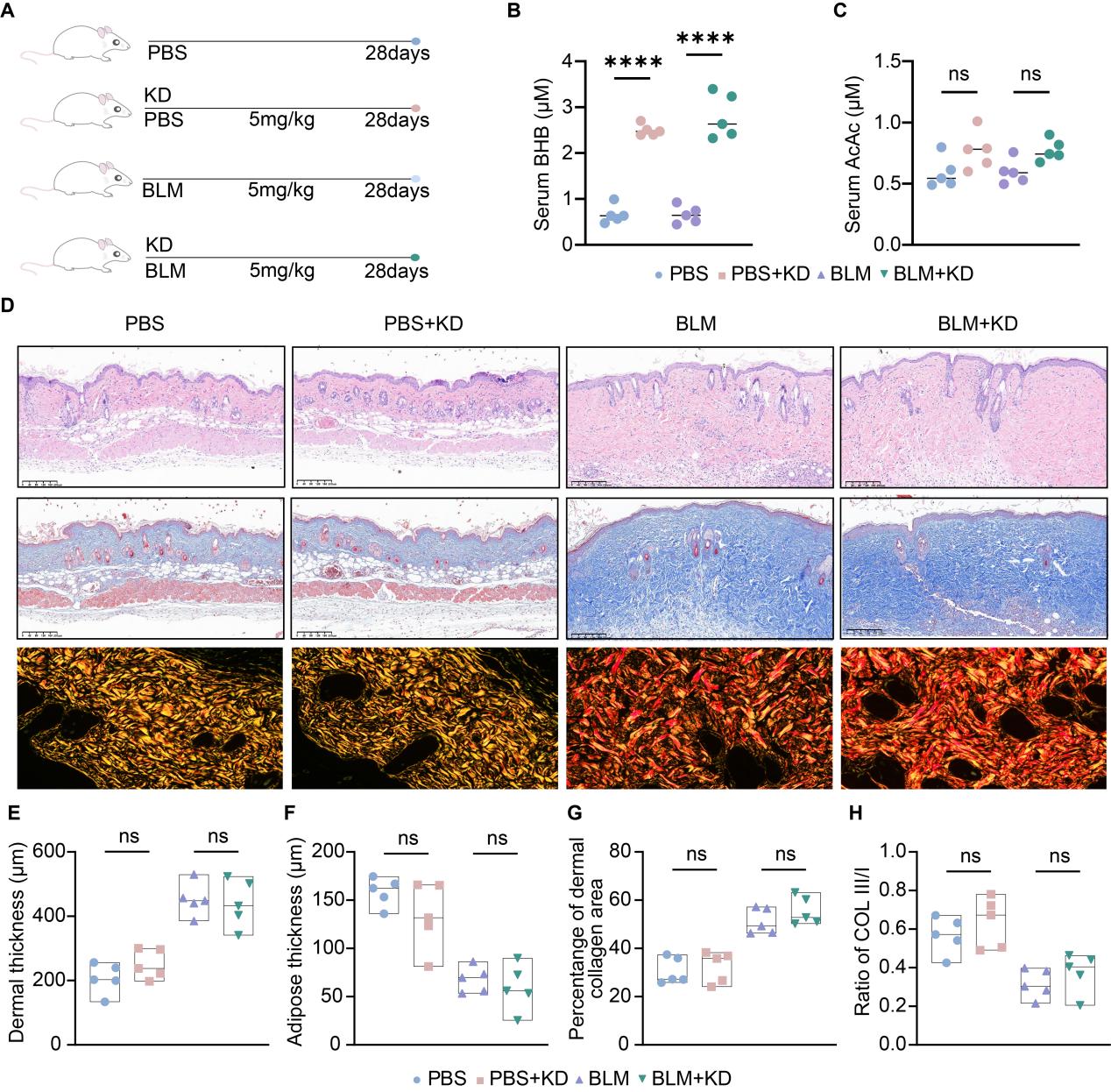


**Fig. S4.2 The ketogenic diet has no significant effect on the skin fibrosis induced by bleomycin.**

(A)Experimental design for ketogenic diet on BLM-treated skin. (B,C)BHB and AcAc levels in whole blood of mice received normal or ketogenic diet for 28 Days. (D) H&E, Masson’s trichrome and Sirius red staining of bleomycin treated skin with or without ketogenic diet. Scale bar, 200 µm. (E-H) Quantitive analysis of dermal(E) and adipose thicknesses(F), collagen coverage(G), and the ratio of type III/I collagen(H) in skin using ImageJ. Statistical significance was analyzed using one-way analysis of variance and two-way analysis of variance with multiple comparisons. * *p* < 0.05, ** *p* < 0.01, *** *p* < 0.001, **** *p* < 0.0001.


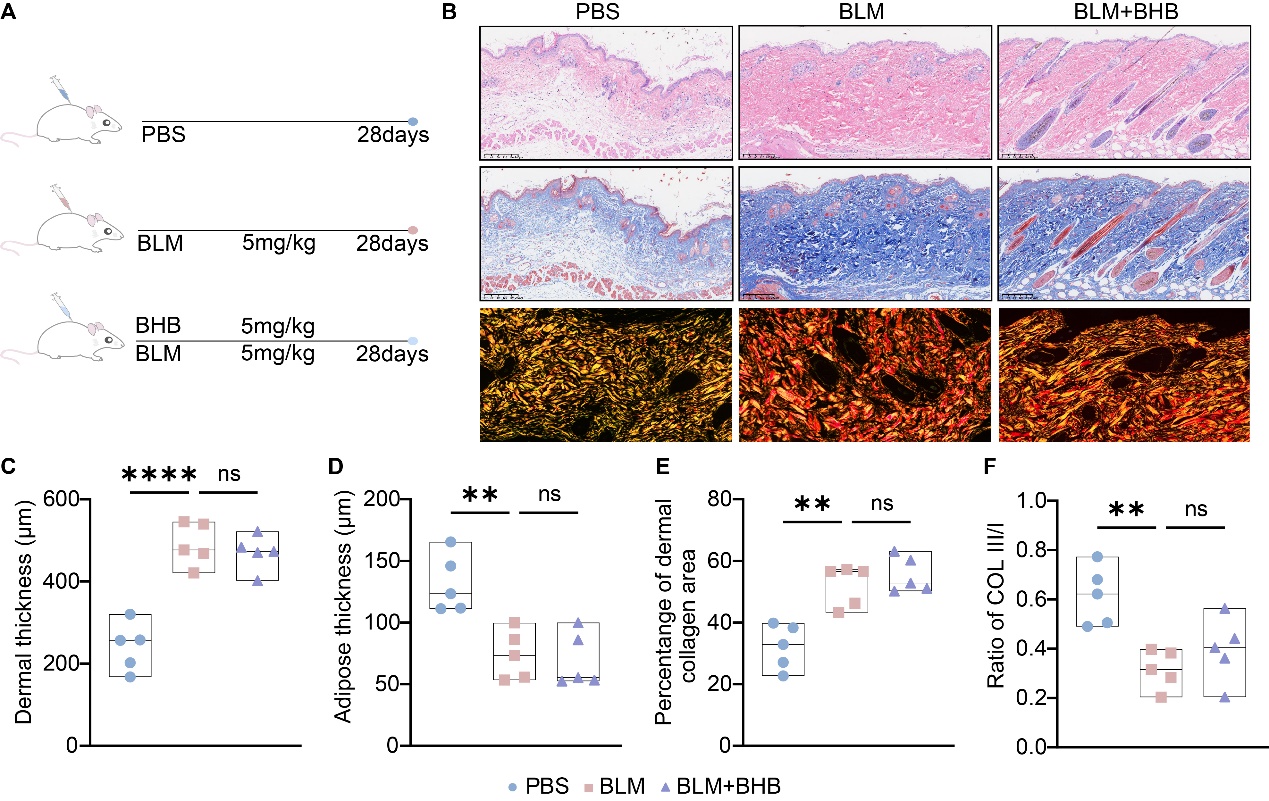


Fig. S4.3 The exogenous BHB injection has showed no significant therapeutic effect on the skin fibrosis induced by bleomycin.

(A)Experimental design for fibrotic examination in subcutaneous injected detecting the role of BHB and BLM mice. (B) H&E, Masson’s trichrome and Sirius red staining of bleomycin treated skin with or without BHB injection. Scale bar, 200 µm. (C-F) Quantitive analysis of dermal(C) and adipose thicknesses(D), collagen coverage(E), and the ratio of type III/I collagen(F) in skin using ImageJ.Data expressed as the mean ± SEM. Statistical significance was analyzed using one-way analysis of variance and two-way analysis of variance with multiple comparisons. * *p* < 0.05, ** *p* < 0.01, *** *p* < 0.001, **** *p* < 0.0001.


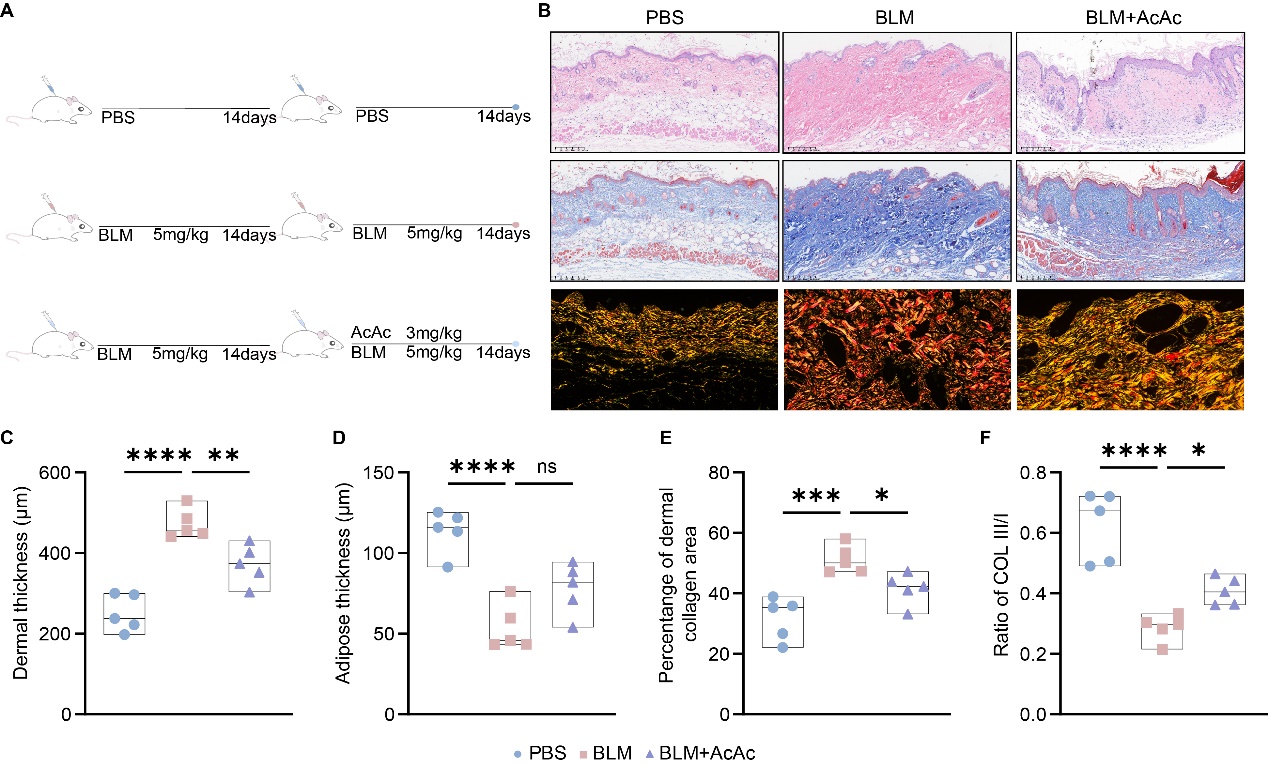


**Fig. S4.4 AcAc treatment partially reversed skin fibrosis induced by bleomycin.**

(A)Experimental design for impact of AcAc on BLM treated skin. (B) H&E, Masson’s trichrome and Sirius red staining of bleomycin treated skin with or without AcAc administration beginning at the 14th days after BLM treatement. Scale bar, 200 μm. (C-F) Quantitive analysis of dermal(C) and adipose thicknesses(D), collagen coverage(E), and the ratio of type III/I collagen(F) in skin using ImageJ. Statistical significance was analyzed using one-way analysis of variance and two-way analysis of variance with multiple comparisons. * *p* < 0.05, ** *p* < 0.01, *** *p* < 0.001, **** *p* < 0.0001.
